# Supplementary material for: Implementation of Financial Incentives for Successful Smoking Cessation in Real-Life Company Settings: A Qualitative Needs Assessment among Employers
Source: Int J Environ Res Public Health. 2019 Dec 16;16(24):5135. doi: 10.3390/ijerph16245135 (PMC6950050; doi:10.3390/ijerph16245135)
Supplement: Supplementary file 1 [file ijerph-16-05135-s001.pdf]

Interview Guide

Note: Questions were slightly adapted based on whether the respondent had experience with smoking cessation group trainings with financial incentives; respondents without previous experience received questions a hypothetical situation in which group trainings and incentives would be implemented.

Background employer

• What is your age, education and position?
• What type of company do you work for? In which industry?
• What is the background of the company's employees (number of employees, level of education, number of employees with low socioeconomic status, extent of absenteeism, smoking behavior)?
• Does the company where you work have a policy on lifestyle or healthy behavior? If so, what is the content of this in brief?
• What is your responsibility in deciding whether or not to offer stop smoking training to employees? Who else is responsible?

General

• When did your company offer the stop smoking training (in combination with financial incentives)?
  o How did this go (broadly)? What went well? What did not go well?

Reach

• How did the distribution of the invitation to participate in the stop smoking training (including rewards) to employees go? Which approach was used for this? How did this approach come about? What went well and less well?
• Were all employees (both executive staff and management) aware of the stop smoking training? Which employees do or do not? What is the deliberate choice to inform some employees or not? If yes, why yes / no?
• How many employees ultimately took part in the training? Which employees did or did not? Was this expected? Why not?

Adoption

• What are the most important goals for your company when offering stop smoking training with rewards to employees (for example, healthier employees, higher productivity, less absenteeism)?

• To what extent was there support within the company for offering the stop smoking training (with rewards)? Have measures been taken to increase support (both among executive staff and management)? If yes which one? Which were particularly successful? And which less successful?

• What hindering factors are there for offering stop smoking training (with financial incentives) in this company? What disadvantages are there for the employer to offer such a training?

• What would make it easier to offer stop smoking training (with financial incentives) at this company? What are the benefits of employing such training for employers?

• What was a decisive factor for your company in choosing to offer the stop smoking training?
• What would be a deciding factor to offer financial incentives for quit success in addition to the stop smoking training?

• Do you see offering stop smoking training as a task or responsibility of the employer?
• Do you see offering a financial reward for successful stoppers as a task or responsibility of the employer?

Implementation
• To what extent has the stop smoking training been offered within your company as originally intended? What was done differently than was actually intended? Why? Which factors played a role in this?
• How would you do it if you had to pay the financial incentives as an employer yourself? Where or from whom in the organization do you expect resistance or support? What kind of resistance or support do you expect?
  o What do you think is a reasonable financial reward (suitable for the person, feasible for the organization)?
  o In what form would you like to give a financial reward (voucher, cash, something else)?

Maintenance
• Is the stop smoking training (including financial incentives) currently being offered to employees within your company?
• Do you intend to continue to do this or to do so in the future? What are barriers and facilitators for this?
• What expectations do you have about the (permanent) implementation of a stop smoking training in combination with financial incentives in the future?
• What role does financing the stop smoking training and the financial incentives play in the implementation?

Smoke-free policy
• Do you or your company have intentions or plans to turn the company into a smoke-free environment? Why or why not? If so, which plans?

Remaining
• Do you have anything to add to the interview?
